# Can sheep help to improve positive emotions, mindfulness, and self-efficacy expectancy? A pilot study of animal-assisted intervention as an enhanced CBT-based therapy for substance use disorders

**DOI:** 10.3389/fpsyt.2024.1432679

**Published:** 2024-10-15

**Authors:** Petra Schmid, Carmen Nauss, Claudia Jauch-Ederer, Petra Prinz, Stefan Tschöke, Carmen Uhlmann

**Affiliations:** ^1^ Klinik für Psychiatrie und Psychotherapie I, Universität Ulm, Ulm, Germany; ^2^ Zentrum fuer Psychiatry Suedwuerttemberg, Versorgungsforschung, Ravensburg, Germany; ^3^ Prinzenhof, Leutkirch, Germany

**Keywords:** animal-assisted intervention, sheep, psychiatric, inpatient, addiction, emotion, substance use disorder

## Abstract

**Introduction:**

Substance use disorders (SUDs) are common, and there is evidence of clinically significant benefit of cognitive behavioral therapy (CBT). The efficacy of CBT in SUDs has been confirmed, although relapse rates of 40%–60% have been reported. An enhancement of CBT-based therapy through an animal-assisted intervention (AAI) with sheep to normalize the occurrence of negative emotions and improve positive emotions as well as mindfulness and self-efficacy expectancy was investigated.

**Methods:**

A single-session AAI with sheep in a group setting was investigated against treatment as usual over time. N = 36 psychiatric inpatients with SUDs were examined by questionnaires before and 1 week after the intervention and additionally immediately after the intervention.

**Results:**

Positive emotions improved significantly in the AAI group 1 week after the intervention with a medium effect size, but not in the control group. Similarly, mindfulness and self-efficacy expectancy improved over time in the AAI group. When exploratory results were evaluated immediately after the intervention while still on the farm, the effects in favor of AAI were even larger.

**Conclusions:**

AAI can thus be considered effective in improving positive emotions, mindfulness, and self-efficacy expectancy. The impressive effect sizes immediately after the intervention encourage us to consider what can be done to maintain these even greater effect sizes over time.

**Clinical Trial Registration:**

https://drks.de/search/de/trial/DRKS00027539, identifier DRKS 00027539.

## Introduction

Substance use disorders (SUDs) have a high prevalence worldwide (1). Men are 1.5 to 2.3 times more likely to be affected than women (2). Biological (genetics and developmental stages of the brain) and social (adverse childhood experiences, high stress levels, easy access to drugs, and low social support) factors are recognized as contributing to vulnerability or resilience against the development of SUDs. With regard to the treatment of SUDs, there is evidence of clinically significant benefit of behavioral therapies (1). Cognitive behavioral therapy (CBT) is based on the assumption that behaviors, including substance use, are learned. Through reinforcement processes, the neurobiologically determined rewarding properties of substances (mediated in particular by dopamine) are associated with previously unconditioned stimuli so that a consumption behavior arises and is later maintained (for a detailed overview, see 1). CBT aims to interrupt these learned associations to reduce the likelihood of substance use, manage its consequences, and intervene quickly in the event of relapse (1, 3, 4). This may be achieved by promoting awareness of behavioral patterns and providing the patient with a set of coping skills to functionally regulate negative, as well as positive emotions (1, 3). Self-efficacy, defined as confidence in one’s ability to resolve situations by applying one’s skills (5), increases the likelihood of applying acquired skills and thus also reduces the likelihood of substance use. The efficacy of CBT in SUDs has been confirmed (6), although relapse rates of 40%–60% have been reported (7). Some shortcomings of CBT in SUDs are described. First, CBT focuses heavily on avoidance goals (e.g., risk situations) rather than developing approach-based goals with patients (8). Second, CBT usually works on affect regulation with the goal of cessation of negative emotional states instead of normalizing the occurrence of negative emotions (8) and fostering positive emotions. These may be the key issues for therapeutic success, as SUDs are associated with high levels of negative emotionality and dysfunction in emotion regulation (9), possibly due to traumatic experiences prior to SUDs. For this reason, a non-judgmental mindful perception and acceptance of negative emotions and at the same time an activation of positive emotions is needed to improve treatment of SUD. This could be achieved with an approach based on mindfulness. Mindfulness is defined as an intentional, conscious focus on the immediate, present perception (not on the past or future), which is non-judgmental with regard to thoughts and feelings and is characterized by openness and curiosity (10, 11). In particular, the ability to adopt a non-judgmental and non-reactive attitude toward experiences proved to be a decisive factor for a positive correlation between mindfulness and functional emotion regulation (12). Also, increasing self-efficacy expectations is an important factor in SUD treatment (13). The processes described above could be achieved, for example, through animal-assisted interventions (AAIs) (14). AAI includes interventions that involve animals to positively impact human health and wellbeing (15). AAIs comprise both animal-assisted therapy (AAT) and animal-assisted activity (AAA) (16). AAAs are defined as informal human–animal interactions and interventions conducted by human–animal teams that are goal oriented for motivational, educational, and recreational purposes. AATs, in contrast, are also goal oriented but comprise structured and individualized therapeutic interventions. They are often delivered or directed by licensed healthcare professionals as part of a treatment process (16).

Several reviews on the efficacy of AAI are already available (17–21). They report improvements in positive emotions, social behavior, and level of functioning, while agitated behavior, negative emotions such as anxiety, or clinical symptoms such as depression can be reduced (17, 19, 20). In contrast, reviews criticize both the heterogeneity of the included studies and methodological shortcomings, such as small samples, lack of randomized controlled trials (RCTs), lack of standardization or manualization of interventions, and use of non-specific outcome measures (17, 18, 20). Thus, although there is currently a consensus on the efficacy of AAIs in healthcare, it is not possible to speak of existing evidence (22), and also specific and non-specific factors of AAI have not yet been identified (23).

Therefore, regardless of the reviews, it is worth taking a closer look at single empirical studies with high research standards. In one study, an RCT of n = 61 depressed patients with comorbid child trauma and the effects of a mindfulness-based AAI with sheep was conducted (14). The treatment-as-usual (TAU) group underwent guideline-oriented treatment, and the AAI group received additionally a total of eight manualized animal-assisted sessions in a group setting over an 8-week period. AAI proved to be feasible, highly acceptable, and more effective than treatment as usual in preventing relapse after 1 year; however, statistical significance was scarce. A second AAI study explored the effect of the presence or absence of a therapy dog in the daily routine of inpatients with SUDs and comorbid mental disorders on social interaction as well as on positive and negative emotionality in a control group design. Significant differences in favor of the AAI were found in both the variables improvement of social interaction and emotionality (3). Critically, it is worth noting that there was no standardized procedure for the AAI intervention. Finally, in a series of studies with a pre–post crossover design, the effect of a single-session AAI intervention with a dog was examined. A significant reduction of negative emotions (anxiety) in severely mentally ill inpatients was demonstrated (24–26). Taken together, a group design with a single-session AAI intervention may be useful in improving emotionality in severely mentally ill patients. Due to their genetics, social structure, and sensitivity, sheep have excellent abilities to be used in the field of AAI. They are herd animals and therefore have social behavior skills. Humans can be integrated into their social structure if the sheep are given the opportunity to approach slowly. They provide security and relaxation, as they are very gentle and calm. Due to their sensitivity, mindfulness is necessary in dealing with sheep (27).

The aim of the present pilot study was to examine whether a single session of AAI in a group setting with sheep can reduce negative emotions and improve positive emotions as well as mindfulness and self-efficacy expectancy. For this purpose, psychiatric inpatients with SUDs and comorbid mental disorders were studied directly before and 1 week after the intervention.

## Materials and methods

### Study design

A controlled, repeated-measures trial was conducted between January 2022 and March 2023 comparing TAU with a single-session animal-assisted intervention in addition to TAU (AAI) in inpatients with SUDs. Allocation to the control group (TAU) or AAI group was determined by the timing of inpatient treatment, as AAI sessions were scheduled in advance. To minimize selection bias due to the lack of an RCT design, exactly the same inclusion and exclusion criteria were applied to the AAI and TAU groups.

Ethical approval was obtained from the ethics committee of the University of Ulm (no. 13/22), including approval of the General Data Protection Regulation EU (GDPR) 2016/679. In accordance with the Declaration of Helsinki, participants were informed and provided written informed consent. The study was registered with the German Registry for Clinical Studies (DRKS00027539, date of first registration March 3, 2022).

### Participants

Participants consisted of inpatients in a specialized ward for patients with SUDs and comorbid disorders in a hospital for psychiatry and psychotherapy in Germany. The patients in this ward had already completed the first phase of withdrawal treatment and received further CBT-based psychotherapy. Comorbid diagnoses cover the entire spectrum of mental illnesses. However, patients with comorbid personality disorders, trauma disorders, or attention-deficit/hyperactivity disorder (ADHD) were mainly represented, followed by patients with depression, anxiety, eating disorders, and even schizophrenia.

Inclusion criteria were age between 18 and 65 years, main diagnosis of SUDs, inpatient treatment for at least seven more days, capability for providing consent, and physical requirements such as standing and walking securely. Exclusion criteria were the presence of animal phobia, allergies, and aversion to specific animals (26). Patients in an acute mental crisis were also unable to participate, as were patients with insufficient language comprehension or mental retardation.

### One Health Framework

The One Health Framework is an approach addressing human, animal, and environmental health (28). Quality standards for the use of animals in AAI are available (29–33) and also a risk assessment tool for (canine) assisted interventions (34). These standards were adopted.

The AAI took place at the Prinzenhof and was carried out by two certified professionals. PP is a specialist in animal-assisted education and support [certified International Society for animal assisted Therapy (ISAAT)] and the owner of the sheep; CN is a specialist in animal-assisted interventions (ISAAT) and a nurse and knows the participants from the clinical setting. The breed of the sheep was mountain sheep mix or Coburg fox sheep mix. They were partly bottle-fed and were therefore very people-oriented and trusting. The sheep live together as a flock at the Prinzenhof and were looked after and trained by PP. Sheep are animals that normally flee from predators and therefore also often from humans. The animals in this study were well accustomed to humans and did not show pronounced retreat behavior.

Before starting the intervention, the participants were supervised about the rules for handling sheep and the individual characteristics of the sheep to be taken into account. Participants were also informed that the intervention would be stopped immediately if there was a risk of the sheep’s welfare being compromised. In addition, a so-called “protected” area was available to the sheep when working in either the open stable or the paddock. The sheep were conditioned to go to this area whenever they no longer wanted to participate in the intervention. Study participants were instructed not to enter this area and to respect any retreat behavior of the sheep. During the intervention, PP was primarily responsible for the care and supervision of the sheep and CN for the study participants.

### AAI procedure

The AAI procedure lasted approximately 5 hours including travel time and took place at the farm “Prinzenhof” in Leutkirch, Germany (see also (35)). The additional costs per intervention group include a fee of 200 € for the “Prinzenhof”, the costs for two professionals for 5 hours, and fuel costs. In case of good weather, the intervention was carried out in the sheep paddock, and in case of bad weather, in the open stable. Each AAI group consisted of four participants and four sheep. The AAI procedure was manualized and divided into seven sections.

*1. Observation of the farm owner’s interaction with the sheep:* The farm owner (PP) was observed interacting with the sheep in the separated sheep paddock or the open stable. Her interaction with the animals served as a model of mindfulness and respect for the basic needs of the sheep.
*2. Introducing the sheep*: Still separated by a fence, the sheep were presented individually with names and specific characteristics. This made it easier for the participants to establish a connection with the sheep.
*3. Approach via feeding:* The sheep were fed by the participants over the fence, and the first physical contact took place.
*4. Approach via presence*: The participants sat down on prepared logs/chairs in the paddock/open stable. Contact was established exclusively starting from the sheep moving freely. If a sheep joined a participant, it was allowed to make physical contact by petting, although the decision about the duration of the physical contact was up to the sheep. The persistence of contact was closely associated with the participants’ mindfulness of the sheep’s needs. In most cases, the partnerships between sheep and humans were established for the entire session at this stage on the initiative of the sheep.
*5. Experiencing competence and attachment:* The sheep were leashed by the respective participant, which represented a challenge, even if these sheep were used to it. The sheep then were led out of the paddock/out of the stable and a distance of approximately 200 m was walked together.
*6. Free walk-in mindful interaction:* Afterward, the leash was removed, and the sheep walked the rest of the course together with the participants. Sheep and participants formed a common flock, which allowed them to experience the connection between sheep and humans. The participants were given the opportunity to interact with the sheep and experience the trust that was built up between them.
*7. Farewell:* The sheep were returned to the paddock/stable, given water, and were farewelled by the participants. Over snacks and drinks, the participants conducted their feedback session.

### Materials

As a primary outcome, the *State-Trait Anxiety Inventory—state version* (STAI-S (36)) was used. The test quality criteria, such as internal consistency, validity, and test–retest reliability, were satisfactory (Cronbach’s alpha = .90). The questionnaire consists of 20 four-point Likert-scaled items (not at all, somewhat, moderately, and very much so). All items related to the absence or presence of anxiety, with 10 items representing positive emotions, e.g., “I feel secure”, and 10 items representing negative emotions, e.g., “I am worried”. To differentiate between these two expressions, a sum score for positive and one for negative emotions were calculated for the primary outcome.

For secondary outcomes, the *Freiburg Mindfulness Inventory short version* [FMI (37, 38)] was used. It comprises 14 items, which are assessed on a 4-point Likert scale (almost never, rarely, relatively often, and almost always). The items are worded positively and negatively. The measure has satisfactory test quality criteria. For measuring self-efficacy expectations, the *General Self-Efficacy Expectancy Scale*, German version [SWE (39)] was applied. The questionnaire consisted of 10 items with agreement from 1 to 4, resulting in a sum score from 10 to 40 (40). Good psychometric proprieties were reported (e.g., Cronbach’s alpha = .92).

The SCL-K-9 (41) as the *short version of the Symptom-Checklist* [SCL-90-R (42)] was applied to assess the subjectively perceived symptom burden. The nine items were rated on a 5-point Likert scale according to symptoms in the last days. The SCL-K-9 is suitable as a screening instrument for the assessment of a wide range of psychopathological symptoms.

The *Objective Social Outcome Index* [SIX (43)] was used to assess social integration. The SIX consisted of four items: employment, accommodation, partnership/family, and friendship. The resulting score ranged from 0 to 6. Age, sex, diagnoses, and duration of inpatient stay data were extracted from the medical records after discharge.

Data on sociodemographic variables, degree of social integration, and clinical symptoms were collected to describe and compare the two study groups.

As a qualitative method, the participants were asked to freely write down ideas on the following question: *What did the animal-assisted intervention accomplish?*

The data were collected before the intervention (PRE) and 1 week after the intervention (POST). At PRE, the STAI-S, FMI, SWE, SCL-K-9, SIX, and sociodemographic data were collected. At POST, the STAI-S and FMI were SWE were measured again. Additionally, some questionnaires were given directly after the intervention while still on the farm (INT). Every participant first wrote down his/her ideas on the following question: *What did the animal-assisted intervention accomplish?* Afterward, the STAI-S, FMI, and SWE were presented.

### Power calculation

For AAIs in psychiatric samples, effect sizes of d = .457 (26) and d = .869 (25) were reported for STAI-S over time. According to the case number calculation with G*Power 3.1 and based on the averaged effect size of both reported studies (d = .66), α = 0.05, β = 0.80, one-sided testing and calculating t-test for dependent samples, n = 16 participants per group with full dataset were targeted. To compensate for possible drop-outs, n = 38 participants were to be recruited.

### Statistical analysis

The analysis was performed using IBM SPSS 27^®^. Dichotomous variables were evaluated with the Chi^2^ test. Normal distribution was tested using the Kolmogorov–Smirnov test. Group differences were analyzed for normally distributed data with t-tests for independent groups. Variance homogeneity was analyzed using Levene’s test. For non-normally distributed data and ordinal scaled variables, group differences were tested using the Mann-Whitney U test.

To examine improvements in the two primary outcome sum scores of the STAI-S (negative and positive emotion) over time (PRE and POST), Wilcoxon tests were calculated separately for the AAI and TAU groups. In case of significant improvements observed in Wilcoxon tests, r resp. Cohen’s d was calculated for effect size, with r = .1 resp. d = .2 representing a small effect, r = .3 resp. d = .5 representing a medium effect, and r = .5 resp. d = .8 representing a large effect (44). To examine the between-groups effects, Mann–Whitney U tests were calculated. Due to the significant improvements in AAI in contrast to the TAU group between PRE and POST, the results directly after the intervention (INT) were included in the analysis. Here, the Wilcoxon test (PRE vs. INT) and Mann–Whitney U test (AAI vs. TAU) were calculated. In order to consider all three times (PRE, INT, and POST) in one analysis, a repeated-measures ANOVA was calculated. As Field (45) points out, even if the normal distribution assumption is violated (at INT in this case) or if the assumption of homogeneity of variance is violated (at PRE and POST in this case), ANOVA is considered to be a robust procedure and can therefore be used. The assumption of sphericity was checked using Mauchly’s test of sphericity and was not violated.

For secondary outcomes, the assumption of homogeneity of variance was violated at SWE POST, FMI PRE, and POST; hence, corrections were applied. t-Tests for independent resp. dependent samples were calculated, as well as repeated-measures ANOVAs.

For qualitative analysis of participants’ statements to the question “What did the animal-assisted intervention accomplish?” at INT, they were then analyzed in content using an alternating inductive and deductive procedure. The principle of openness prevailed. The statements relevant to the research purpose were paraphrased and then coded. The codes were then summarized into main and subcategories by consensus. The main categories and the identified subcategories as well as corresponding examples were presented.

## Results

### Comparability of groups

**Figure 1:**
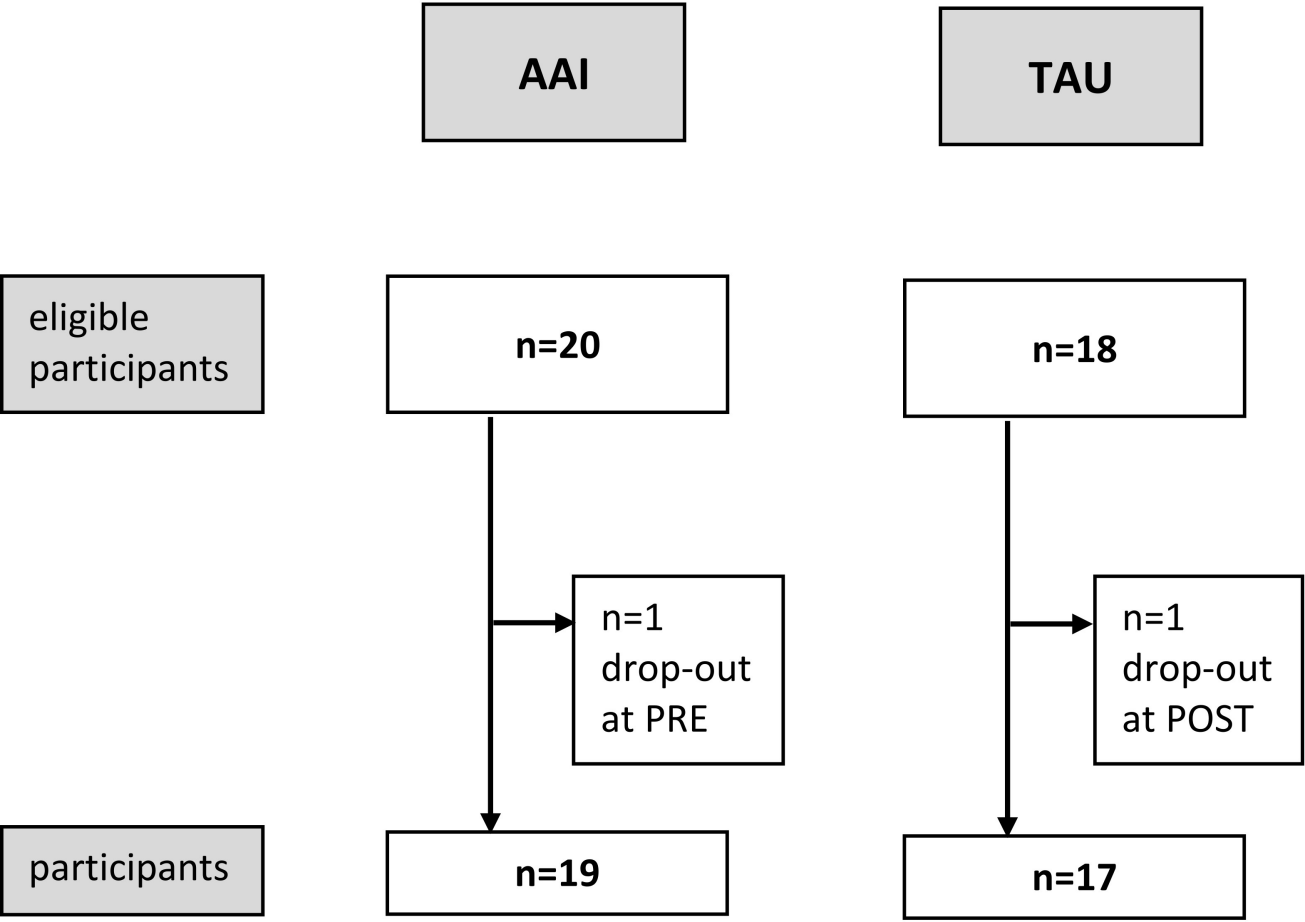
CONSORT for recruitment of both groups, animal-assisted intervention (AAI) and treatment as usual (TAU).

Of the n = 38 recruited patients, n = 20 were assigned to the AAI group and n = 18 to the TAU group. After excluding participants due to missing data, n = 19 participants in the AAI group and n = 17 participants in the TAU group were included in the analyses (see **Figure 1**). The groups did not differ significantly in age [t (34) = −0.871; p >.05], gender [Chi^2^(1,36) = 0.000; p >.05], main diagnosis [Chi^2^(3,36) = 1.730; p >.05], number of secondary diagnoses (U = 160.50; p >.05), and length of stay (U = 143.50; p >.05) in social integration, measured by the SIX (U = 127.00; p >.05), or in subjectively perceived symptom burden, measured by SCL-9-K [t(27.739) = 0.236; p >.05] (see **Table 1**).

**Table 1:** Group comparison of animal-assisted intervention (AAI) and treatment as usual (TAU).

			AAI (n = 19)		TAU (n = 17)		p
Age		M (SD)	45.58	(14.51)	41.35	(14.56)	n.sign.^1^
Female		n (%)	9	(47.4%)	8	(47.1%)	n.sign.^2^
Main diagnosis: dependence syndrome	Alcohol	n (%)	14	(73.7%)	11	(64.7%)	n.sign.^2^
	Opioids	n (%)	1	(5.3%)	2	(11.8%)	
	Cannabinoids	n (%)	3	(15.8%)	4	(23.5%)	
	Multiple drugs	n (%)	1	(5.3%)	0	(0.0%)	
Number of secondary diagnoses		M (SD)	5.05	(2.37)	5.53	(3.50)	n.sign.^3^
Duration of stay (in days)		M (SD)	39.74	(18.86)	33.41	(7.57)	n.sign.^3^
Social integration (SIX)		M (SD)	2.84	(1.74)	3.47	(1.59)	n.sign.^3^
Symptom burden (SCL-9-K)		M (SD)	1.93	(1.13)	2.00	(0.59)	n.sign.^1^

SIX, Objective Social Outcome Index; SCL-9-K, subjectively perceived symptom burden.

^1^t-Test for independent groups.

^2^Chi^2^ test.

^3^Mann–Whitney U test.

### Primary outcome

**Figure 2:**
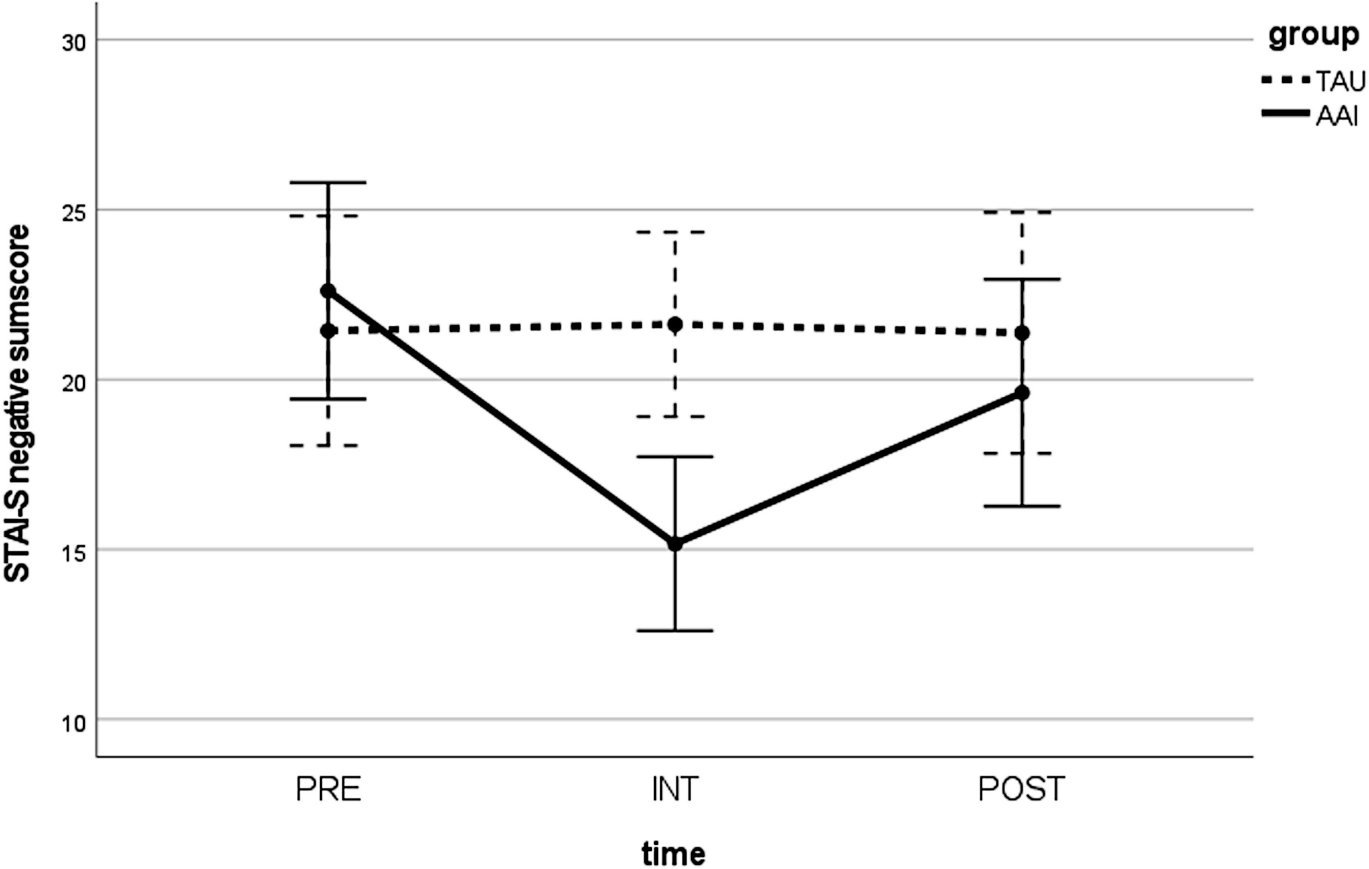
Values in the STAI-S positive emotion sum score over time (PRE, INT, and POST) for both groups (AAI vs. TAU). STAI-S, State-Trait Anxiety Inventory—state version; PRE, pre-measurement before intervention; INT, measurement directly after intervention; POST, post-measurement 1 week after intervention; AAI, animal-assisted intervention; TAU, treatment as usual.

**Figure 3:**
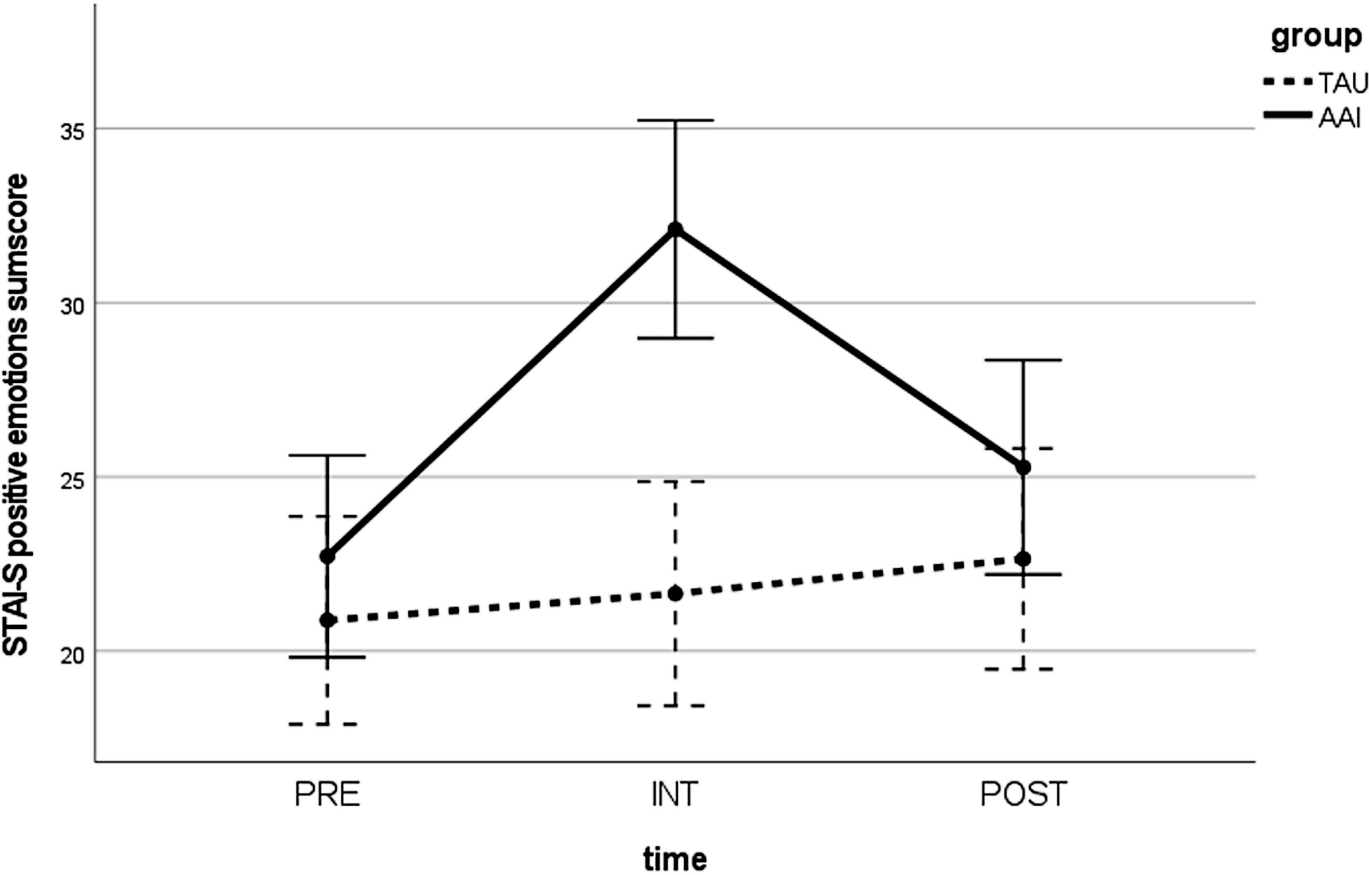
Values in the STAI-S negative emotion sum score over time (PRE, INT, and POST) for both groups (AAI vs. TAU). STAI-S, State-Trait Anxiety Inventory—state version; PRE, pre-measurement before intervention; INT, measurement directly after intervention; POST, post-measurement 1 week after intervention; AAI, animal-assisted intervention; TAU, treatment as usual.

The STAI-S positive emotion sum score displayed a significant improvement over time (PRE and POST) for the AAI group (z = −2.447; p <.05) with a medium effect size (r = .397). However, there was no significant reduction over time in the STAI-S negative emotion sum score (z = −1.724; p = .085). There were no significant differences between the two groups (AAI vs. TAU), neither for PRE (negative emotions: U = 132.500; p >.05, positive emotions: U = 155.500; p >.05) nor for POST (negative emotions: U = 140.000; p >.05, positive emotions: U = 123.500; p >.05). When analyzing the difference between the time before and immediately after the intervention (INT) in the AAI group, the STAI-S negative emotion sum score was significantly reduced (z = −3.336; p <.05) with a large effect size (r = .548), and the STAI-S positive emotion sum score was significantly improved (z = −3.623; p <.001), also with a large effect size (r = .596). At INT, the two groups (AAI and TAU) differed significantly in both sum scores again with large effect sizes (negative emotions: U = 58.000; p <.001; r = .531; positive emotions: U = 39.500; p <.001; r = .634). In repeated-measures ANOVA, a significant time × group interaction was observed for the STAI-S negative emotion sum score [F(2,64) = 6.802; p <.01] and the STAI-S positive emotion sum score [F(2,66) = 11.968; p <.001] (see **Figures 2**, **3**).

### Secondary outcomes

**Figure 4:**
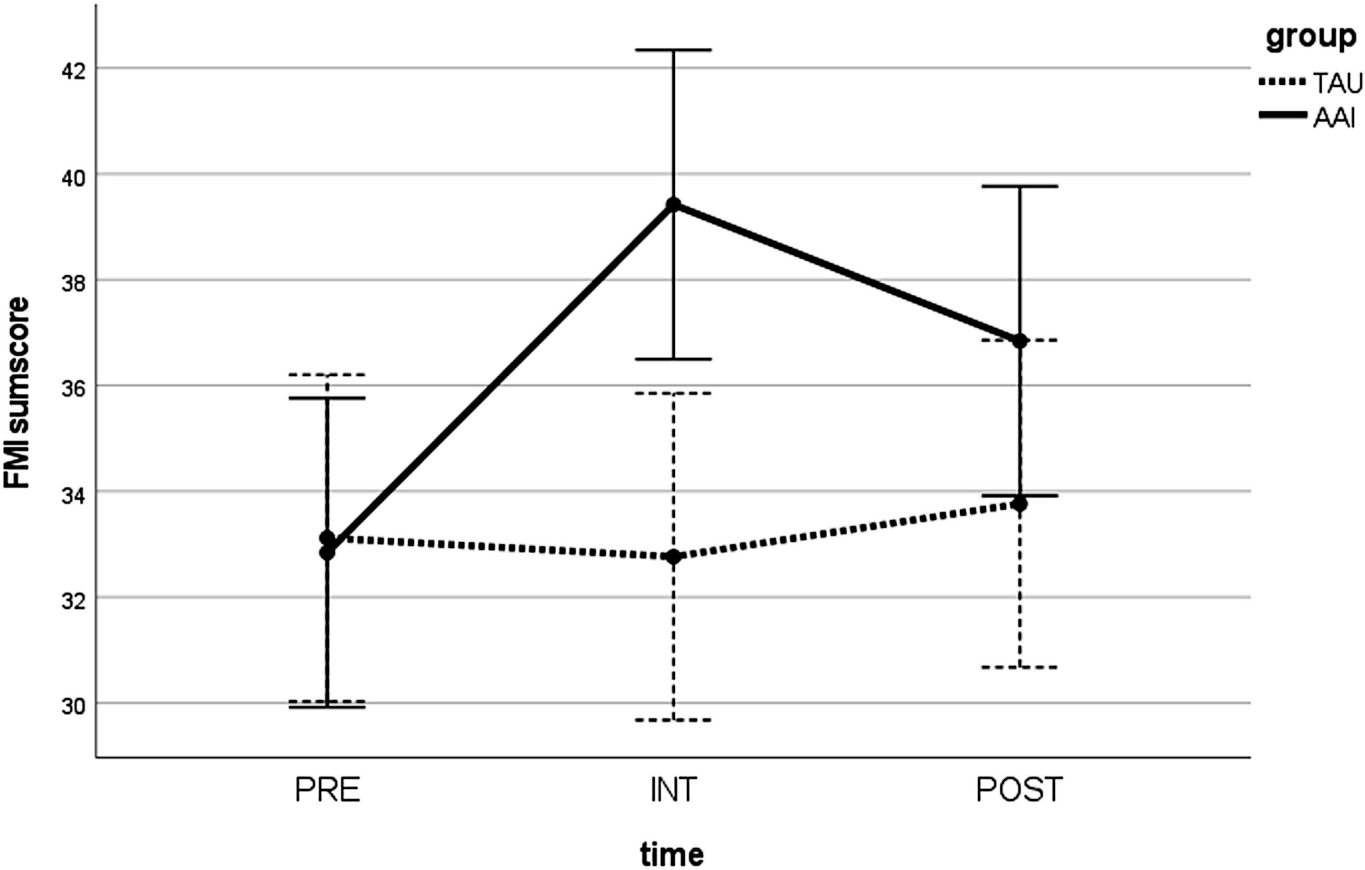
Values in the FMI sum score over time (PRE, INT, and POST) for both groups (AAI vs. TAU). FMI, Freiburg Mindfulness Inventory; PRE, pre-measurement before intervention; INT, measurement directly after intervention; POST, post-measurement 1 week after intervention; AAI, animal-assisted intervention; TAU, treatment as usual.

In the mindfulness score (FMI), the AAI group improved significantly from PRE to POST [t(18) = −3.020; p <.01; Cohen’s d = .637] in contrast to TAU [t(16) = −0.495; p >.05]. The two groups did not differ significantly at either PRE [t(32.212) = 0.134; p >.05] or POST [t(33.922) = −1.1483; p >.05]. When including INT in the analysis, a significant time × group interaction [F(2,68) = 7.261; p <.01] was observed. Both groups showed similar levels of mindfulness at PRE. While the TAU group remained at a stable level of mindfulness throughout, the AAI group improved in their mindfulness at INT, but this effect diminished at POST (see **Figure 4**).

**Figure 5:**
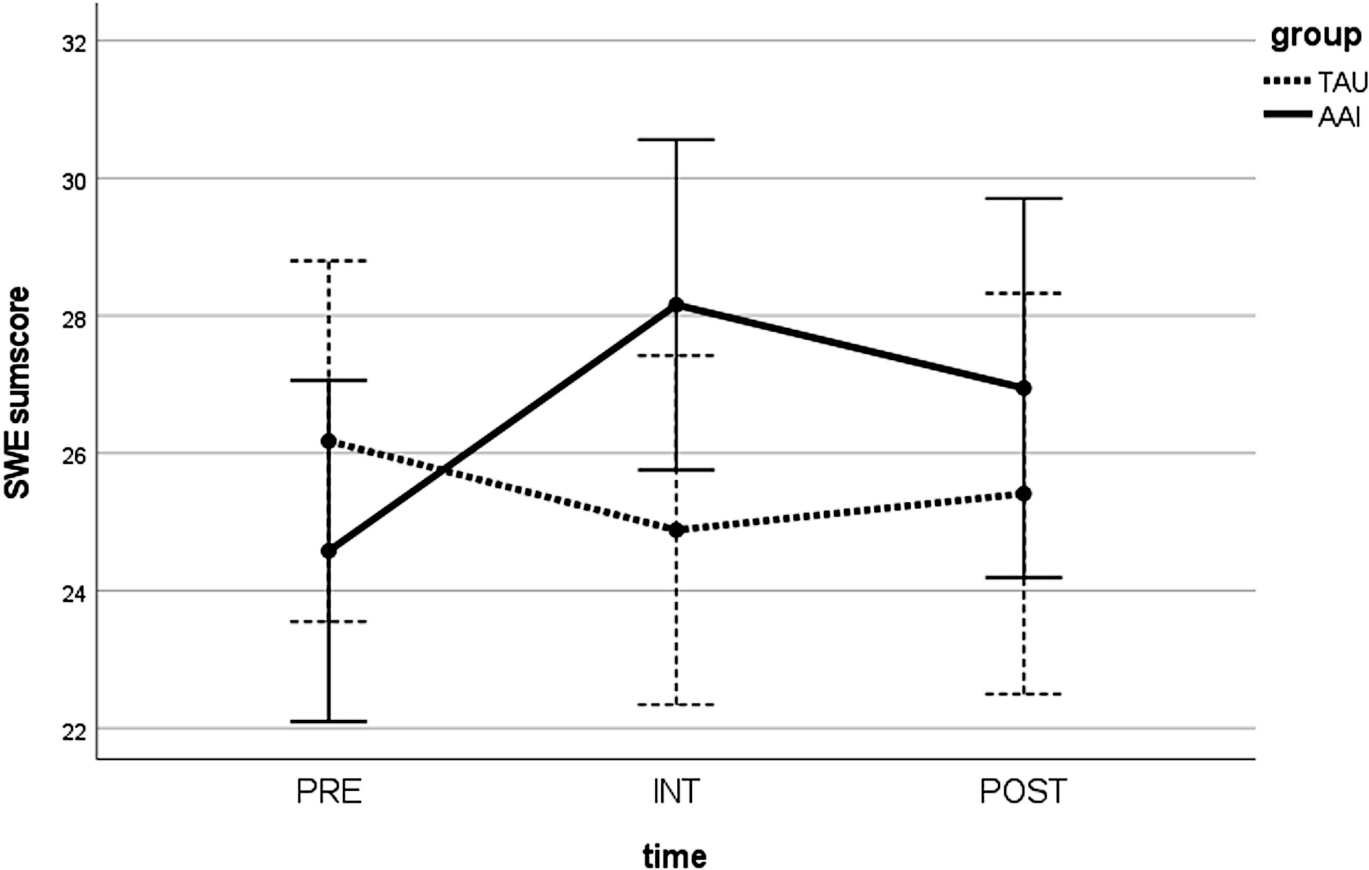
Values in the SWE sum score over time (PRE, INT, and POST) for both groups (AAI vs. TAU). SWE, General Self-Efficacy Expectancy Scale, German version; PRE, pre-measurement before intervention; INT, measurement directly after intervention; POST, post-measurement 1 week after intervention; AAI, animal-assisted intervention; TAU, treatment as usual.

The AAI group demonstrated also significant improvements in self-efficacy expectancy (SWE) at POST [t(18) = −4.095; p <.01; Cohen’s d = .536], while the TAU group did not [t(16) = 0.982; p >.05]. Again, the groups did not differ significantly from each other at either PRE [t(34) = 0.899; p >.05] or POST [t(32.664) = −0.774; p >.05]. Also, a significant time × group interaction resulted [F(2,68) = 9.112; p <.05]. Both groups started at similar levels, with the TAU participants’ scores changing little over time, while the AAI group’s scores showed a peak at INT (**Figure 5**).

### Qualitative analysis of participants’ statements

The n = 78 statements made by the n = 19 AAI participants in response to the question “What did the animal-assisted intervention accomplish?” could be grouped into three categories. As **Table 2** displays, the category “evoking positive valence” was mentioned most frequently, with a total of 53 statements. The participants verbalized mainly to have experienced some kind of mindfulness (n = 18), joy and fun (n = 10), and some sort of closeness (n = 8). The second main category is “decreasing negative valence” with n = 9 statements. The participants reported experiencing reduced rumination, prejudice, fear, and tension. In the last category with n = 16 statements, aspects of “positive valence in interacting with animals and nature” were included.

**Table 2:** Categorized main categories and subcategories from the statements of the n = 19 participants of the AAI group to the question: “What did the animal-assisted intervention accomplish?”.

Main category	Total N	Subcategory	N	Examples
Evoking positive valence	53	Mindfulness	18	Inner peace, calmness, more calm and more confidence, I was able to switch off completely, I feel a deep sense of reassurance, great serenity, clear mind
		Joy and fun	10	I had a lot of fun, great joy, it gave me joy
		Closeness, security, confidence	8	I felt closeness, I felt security, trust, confidence
		Satisfaction	5	Satisfaction, the sheep make me feel very comfortable
		Relaxation	3	I was a little more relaxed than usual, very relaxing
		Positive memories	3	Beautiful memories of my childhood, it was great and reminded me of my earlier career
		Others	6	I perceived my feelings more deeply, it has strengthened my self-esteem, on the whole, it was a great day and, great experience!
Decreasing negative valence	9	Reduced rumination	4	My thoughts and doubts were gone, other thoughts rather good but my thoughts still always wander (but not negatively TODAY)!
		Reduced prejudice	2	I was among people, something I usually avoid; I perceived my fellow patients differently
		Reduced fear	2	I had no fear, I was not afraid of sheep at all
		Reduced tension	1	My tension was less
Positive valence in interacting with animals and nature	16	Calming through animal interaction	6	The animals have something calming, mutual calming of humans and animals, stroking sheep was good
		Connectedness with animal/nature	3	Unity of human and animal, I felt connected with the sheep Toni, 100% closeness to nature
		Others	7	Feeling as if the sheep reflects the human, the sheep were kind and friendly, I have taken the sheep to my heart

## Discussion

The feasibility of a single-session AAI with sheep as an enhanced CBT-based group therapy for SUDs with comorbid disorders was examined. The effect of reducing negative emotions and improving positive emotions as well as mindfulness and self-efficacy expectancy was investigated. In n = 36 participants, the primary outcome STAI-S positive emotions revealed a significant improvement with a medium effect size for the AAI group (r = .397). No effect was observed in the control group with treatment as usual. Thus, our AAI-enhanced CBT treatment was effective in improving positive emotions, which was still measurable 1 week after the intervention (POST). This is consistent with previous studies examining the effectiveness of AAI in changing emotionality in only a single session (24–26). In these studies, the effects were measured before and immediately after the intervention. For this reason, in addition to the reported results between PRE and POST, the effects immediately after the AAI (INT) were also examined. Here, the analyses yielded an even greater effect size for the changes in the positive (r = .596) and negative emotion sum scores (r = .548). In the secondary outcomes, mindfulness (FMI) and self-efficacy expectancy (SWE) also improved significantly between PRE and POST and again even more immediately after the intervention (INT). Taken together, our AAI-enhanced CBT approach seems to have succeeded in activating positive emotions on the one hand and reducing negative emotions on the other hand, both from a mindful, non-judgmental, accepting attitude. It also appears that the participants have succeeded in internally attributing the processes described above and therefore increasing self-efficacy expectancy. In SUDs associated with the presence of overwhelming negative emotions and deficits in emotion regulation (9), this AAI-enhanced CBT approach could help to improve therapeutic outcomes. The advantage could be that our approach promotes positive emotions and normalizes negative emotions instead of focusing only on emotion regulation, as opposed to CBT alone. This is also supported by the results of the qualitative analysis. Participants’ statements about evoking positive valences (e.g., joy, closeness, mindfulness, contentment, and relaxation) and decreasing negative valences (e.g., anxiety, rumination, prejudice, and tension) can be seen in relation to the STAI-S results with its changes in positive and negative emotions.

The challenge for subsequent studies will be how to maintain the large effect seen in our and other reported studies (24–26) immediately after the intervention (INT) over a longer period of time. Schramm et al. (14) conducted therefore one booster session 3 months after finishing their 8-week program. Further studies will have to address the question of how the emotional moment of the AAI, which was experienced directly in the AAI, can be recreated and retrieved later. Imaginative techniques enriched with external representations of the sheep intervention are conceivable here. This could be realized, for example, through a guided imagination about the individual emotional moment of the AAI experience, anchored externally via a piece of sheep wool. Further research is needed to address this point.

Another issue to discuss is the effect of being outdoors in nature and how these circumstances impact participants. Nature-based interventions have proved to increase positive emotions and reduce negative emotions as well as anxiety and depression (46, 47). Our study also demonstrates the importance of the nature-based effect. The third category in the qualitative analysis included statements containing participants’ interaction with animals and nature, which was valued positively. It must therefore ultimately remain open whether the interaction with sheep, the interaction with nature, or a combination of both is the basic principle for the reported results. Subsequent studies will have to investigate this.

There are several limitations. The main limitation is that we were not able to conduct a randomized controlled trial due to organizational feasibility. Instead, we used a non-randomized control group design. The advantage of this approach was the high clinical–ecological validity. An attempt was made to eliminate a possible selection bias by checking the comparability of the two groups. There were no differences in sociodemographic and clinical data, and thus, a comparability of the groups was assumed. However, the risk of a selection bias is high and cannot be excluded. In a subsequent study, the use of an RCT design is therefore strongly recommended, but a blinding strategy would not be possible in a clinical sample where the TAU group receives no further intervention.

A second limitation concerns the duration of the observation period, which was limited to a total of 1 week. This does not allow any statement about the effect of the AAI beyond this period. Even if such clear effects occurred with INT, statements about the further course of therapy and outcome as well as the further disorder progression are not possible. Perhaps ecological momentary assessments (EMAs) would be useful to more precisely capture and monitor the effect detected at INT. This could also happen over a longer period of time.

At last, the generalizability of the results found in our study is limited. The inpatients studied here were a highly selective sample of SUD patients with a high rate of comorbidity, a somewhat impaired level of functioning, and only a short duration of abstinence. Also, AAI results may vary in other farms and other species, such as dogs.

In conclusion, according to empirical studies, CBT has been the treatment of choice for SUDs alongside medication. However, given the high relapse rates of even successfully treated SUD patients, the question arises of how to enrich CBT. The activation of positive emotions and the simultaneous enabling of a non-judgmental perception and acceptance of negative emotions could provide a new treatment ingredient. Our concept of AAI-enhanced CBT follows this approach. As demonstrated, the AAI was successful in reducing negative emotions and improving positive emotions as well as mindfulness and self-efficacy expectancy immediately after the intervention. Unfortunately, the effect could not be fully maintained after 1 week. Nevertheless, the investigated AAI-enhanced CBT approach seems to be beneficial for emotion activation and tolerance, and therefore, it could be useful in the treatment of emotional dysregulation of SUDs, which is to date under too little consideration in pure CBT.

## Data Availability

The raw data supporting the conclusions of this article will be made available by the authors, without undue reservation.
